# Flap Failure and Re-exploration in Head and Neck Free Tissue Reconstruction: The Impact of the Venous System Choice

**DOI:** 10.7759/cureus.88421

**Published:** 2025-07-21

**Authors:** Takuya Suzuki, Yasuhiro Sakata, Shinji Kumegawa, Satsuki Tachibana, Kazuki Ueno, Yoshitaka Wada, Shinichi Asamura

**Affiliations:** 1 Department of Plastic and Reconstructive Surgery, Wakayama Medical University, Wakayama, JPN

**Keywords:** anastomosis, head and neck reconstruction, microsurgery, thrombosis, venous system

## Abstract

Background and objective

Free tissue transfer is widely performed in head and neck reconstruction. However, venous thrombosis at the vascular anastomosis remains a serious complication and a major cause of re-exploration. Hence, we conducted a retrospective case series study to evaluate risk factors for venous thrombosis that induce re-exploration.

Methods

We conducted a retrospective review of 143 flaps for head and neck defects treated at our institution between August 2015 and March 2023. The following patient-related and operative variables were collected from the clinical records: recipient vein, age at the time of surgery, sex, tumor site, flap type, operation time, volume of blood loss, chronic comorbidity, and hematologic status.

Results

Re-exploration due to flap failure was performed in eight cases (5.6%), two of which could be salvaged, and six of which required further reconstruction. The overall flap survival rate was 95.8%. Cases with a single anastomosis to the superficial venous system (SVS) presented a higher risk of re-exploration than those with an anastomosis to the deep venous system (DVS).

Conclusions

Based on our findings, single anastomosis to the SVS, when the internal jugular vein is unavailable, is associated with a higher risk of re-exploration.

## Introduction

Head and neck reconstruction using free tissue was first reported in the 1970s [1], and with the advances in microsurgical techniques, the flap survival rate is now estimated to exceed 95% [2]. While the success rate is favorable, major complications could require further reconstruction with new free tissue, making it key to predict the risk of complications. Venous thrombosis is a major complication in free tissue reconstruction, responsible for more than half of re-exploration cases [3]. While risk factors for venous thrombosis have been widely studied [4], there is no consensus yet. The number of anastomoses [5-11] and the choice of recipient vein [12-15] are issues of particular interest. Multiple venous anastomoses have been identified in several meta-analyses to reduce the risk of flap failure, venous thrombosis, and re-exploration [5-7]. However, some argue against routine multiple anastomoses, citing concerns about decreased blood flow velocity and increased operative time [11].

Selection of the recipient vein is also cited as a variable that could affect the rate of re-exploration. Recipient veins could be classified as either the deep venous system (DVS) or the superficial venous system (SVS). The DVS comprises the internal jugular vein and its branches, and is responsible for venous return from the brain and pharynx. The SVS comprises the anterior jugular vein, the external jugular vein, and the facial vein and is responsible for venous return from the face and oral cavity [16]. The DVS and SVS are sometimes referred to as the internal jugular vein system (IJVS) and external jugular vein system (EJVS), respectively. Some reports have suggested that IJVS is less likely to cause complications [13,14] while others suggest that there is no difference between the venous systems [15]. In this study, we examined the risk factors for re-exploration and thrombosis in terms of anastomotic vessel selection and other operative and patient-related factors.

## Materials and methods

Patients

**Figure 1:**
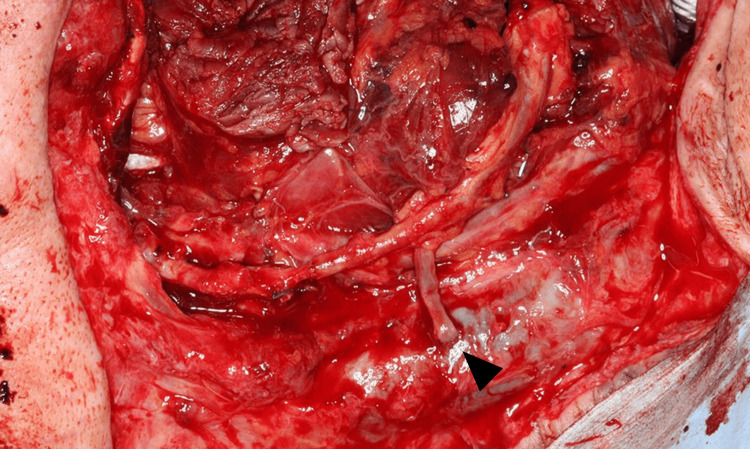
Intraoperative view of a case of anastomose to deep venous system Vein from the anterolateral thigh flap is anastomosed to the internal jugular vein in an end-to-side manner (black arrow)

**Figure 2:**
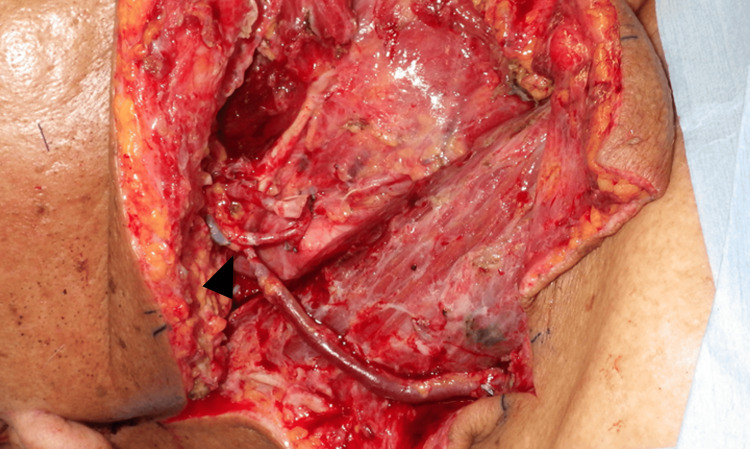
Intraoperative view of a case of anastomose to superficial venous system Vein from the rectus abdominis musculocutaneous flap is anastomosed to the external jugular vein in an end-to-end manner (black arrow)

This study included all patients who underwent free tissue reconstruction of the head and neck region at Wakayama Medical University between August 2015 and March 2023. Patients who underwent reconstruction for tissue defects resulting from trauma, burns, or infection, as well as cases where a local flap was used concomitantly, patients with a follow-up period of less than six months, were excluded. The type of flaps was chosen based on defect size and location, and required pedicle length. The flap survival rate at the six-month time point was used for the analysis. The following patient-related and operative variables were collected from clinical records: age at the time of surgery, sex, BMI, underlying patient factors (diabetes mellitus, smoking), history of preoperative chemotherapy, history of preoperative radiotherapy, hematologic status (hemoglobin, hematocrit, platelet, albumin, total protein), operation time, volume of blood loss, the recipient vein and its system (DVS or SVS), and re-exploration rate. Representative intraoperative views are shown in Figures 1, 2, respectively. All anastomosis was performed using hand-sewn techniques.

Postoperative management

Patients were all administered prostaglandin E1 (120 mg/day) for at least one week postoperatively. The flaps were monitored by the clinician every four hours during the first postoperative week, assessed by the color of the flap, warmth, refill, pin-prick test, and ultrasonography. In case of an abnormal finding on the flap during monitoring, and the cause was suspected to be the site of anastomosis, re-exploration was performed to check the site of anastomosis.

Statistical analysis

Univariate logistic regression analysis was performed to assess the influence of preoperative hematologic status, operative time, and blood loss as potential risk factors for re-exploration. For comparisons of other variables between the two groups, the Mann-Whitney U test was used for continuous variables (age and BMI), and Fisher’s exact test was used for categorical variables (gender,　history of diabetes mellitus, smoking, preoperative chemotherapy, and preoperative radiotherapy). A p-value of less than 0.05 was considered statistically significant. All statistical analyses were performed using JMP Pro 17.2 (SAS Institute Inc., Cary, NC).

Ethical approval

This study has been approved by the Wakayama Medical University Institutional Review Board (Approval number: 3921), and it was performed in accordance with the tenets of the Declaration of Helsinki. The study details were published on the university website for opt-out purposes.

## Results

Patient characteristics and operative factors

Of the 145 cases initially included in the study, 143 were selected for the final analysis, consisting of 118 men and 25 women. The age of patients at the time of surgery ranged between 22 and 85 years (mean age: 63.2 years). A summary of the tumor sites and types of flaps is presented in Tables 1, 2, respectively.

**Table 1:** Tumor site

Site	N (%)
Hypopharynx	36 (25.1)
Tongue	27 (18.9)
Oropharynx	21 (14.7)
Maxilla	19 (13.3)
Mandible	15 (10.5)
Floor of the oral cavity	5 (3.5)
Pharynx	3 (2.1)
Other region	17 (11.9)
Total	143

**Table 2:** Type of flaps ALT: anterolateral thigh; RAMC: rectus abdominis musculocutaneous; SCIP: superficial circumflex iliac perforator; DIEP: deep inferior epigastric perforator; PAP: profunda femoris artery

Type of flap	Number of flaps (%)	Re-explored, n (%)
Free jejunum	41 (28.7)	1 (2.4)
ALT	40 (28.0)	2 (5.0)
RAMC	17 (11.9)	0 (0.0)
Radial forearm	14 (9.8)	0 (0.0)
SCIP	13 (9.1)	3 (23.1)
Fibula	7 (4.9)	0 (0.0)
DIEP	7 (4.9)	1 (14.3)
Scapula	3 (2.1)	1 (33.3)
PAP	1 (0.7)	0 (0.0)

Re-exploration was required in eight cases, among which venous thrombosis was detected in three cases, arteriovenous thrombosis was detected in two cases, and arterial thrombosis was detected in one case. In the remaining two cases, no thrombosis was detected at the anastomotic site, and we were unable to definitively identify other etiological causes. Two of the flaps could be salvaged, but six flaps required further reconstruction. The overall flap survival rate was 95.8%. Clinical features of the re-explored cases are shown in Table 3.

**Table 3:** List of cases requiring re-exploration due to flap failure ^*^IJV double anastomosis Postop: Time from completion of initial surgery to re-exploration; SCIP: superficial circumflex iliac artery perforator; ALT: anterolateral thigh; DIEP: deep inferior epigastric perforator; EJV: external jugular vein; IJV: internal jugular vein

No.	Age, years	Gender	Site of the tumor	Type of flap	Recipient vein	Site of thrombosis	Postop	Salvage result
1	32	Female	Mandible	Scapula	EJV	Arterial anastomosis	7 days	Survived
2	73	Female	Tongue	SCIP	IJV^*^	Arterial/venous anastomosis	5 days	Flap loss
3	49	Female	Tongue	ALT	IJV	Venous anastomosis	3 days	Flap loss
4	68	Male	Maxilla	SCIP	Facial vein	Venous anastomosis	6 hours	Survived
5	56	Male	Oropharynx	SCIP	EJV	Arterial/venous anastomosis	3 days	Flap loss
6	72	Male	Hypopharynx	Jejunum	EJV	Venous anastomosis	15 hours	Flap loss
7	75	Male	Tongue	DIEP	IJV and EJV	Not detected	10 days	Flap loss
8	68	Male	Oropharynx	ALT	IJV and EJV	Not detected	17 hours	Flap loss

No significant differences were found between cases with and without re-exploration regarding patient factors (Table 4), hematologic status (Table 5), and operative time or blood loss volume (Table 6).

**Table 4:** Patient characteristics ^*^Mann-Whitney U test; ^**^Fisher’s exact test IQR: interquartile range (25th-75th percentile)

Characteristics	All (n=143)	No re-exploration (n=135)	Re-exploration (n=8)	Value	P-value
Age, years					
Median (IQR)	67 (56-73)	67 (56-73)	68 (50.8-73)	Z=-0.035	0.97^*^
BMI					
Median (IQR)	20.3 (18.5-23.2)	20.3 (18.4-23.2 )	20.7 (18.5-22.1)	Z=-0.18	0.86^*^
Gender					
Female, n (%)	25 (17.5)	22 (15.4)	3 (2.1)	-	0.14^**^
Male, n (%)	118 (82.5)	113 (79.0)	5 (3.5)		
Diabetes mellitus					
Yes, n (%)	41 (28.7)	41 (28.7)	0 (0.0)	-	0.11^**^
No, n (%)	102 (71.3)	94 (65.7)	8 (5.6)		
Smoking					
Yes, n (%)	104 (72.7)	97 (67.8)	6 (4.2)	-	1.00^**^
No, n (%)	40 (27.3)	38 (26.6)	2 (1.4)		
Preoperative chemotherapy					
Yes, n (%)	85 (59.4)	82 (57.3)	3 (2.1)	-	0.27^**^
No, n (%)	58 (40.6)	53 (37.1)	5 (3.5)		
Preoperative irradiation					
Yes, n (%)	1 (0.7)	1 (0.7)	0 (0.0)	-	1.00^**^
No, n (%)	142 (99.3)	134 (93.7)	8 (5.6)		

**Table 5:** Univariate logistic regression analysis for hematologic status and rate of re-exploration CI: confidence interval

Hematologic status	Total (n=143)	No re-exploration (n=135)	Re-exploration (n=8)	Odds ratio	95% CI	Wald χ²	P-value
Hemoglobin (g/dL)	11.72	11.72	11.74	1.25	0.12–14.68	0.03	0.86
Hematocrit (%)	35.21	35.21	35.18	0.92	0.39–2.07	0.04	0.84
Platelet (×10000/μL)	25.46	25.53	24.21	0.99	0.90–1.08	0.06	0.80
Albumin (g/dL)	3.64	3.64	3.68	1.83	0.23–15.96	0.32	0.57
Total protein (g/dL)	6.38	6.38	6.28	0.65	0.19–2.48	0.41	0.52

**Table 6:** Univariate logistic regression analysis for operative time and blood loss volume CI: confidence interval

	Total (n=143)	No re-exploration (n=135)	Re-exploration (n=8)	Odds ratio	95% CI	Wald χ²	P-value
Operation time (per 60 min)	740.3	736.2	810.3	1.09	0.88–1.27	0.17	0.32
Blood loss volume (per 100 ml)	542.1	543.5	517.6	0.96	0.74–1.12	0.99	0.68

Venous anastomosis

Overall, 74 cases were double venous anastomosis, and 69 cases were single venous anastomosis. Re-exploration was performed in four cases with double anastomosis and four cases with single anastomosis. Among single cases of venous anastomosis, a comparison between the SVS and DVS groups is presented (Table 7), showing that the risk of re-exploration was significantly higher in the SVS group (odds ratio: 21.4, 95% CI: 2.0 to 231.2, p=0.01 by Fisher’s exact test).

**Table 7:** Venous system selection and re-exploration rate in single anastomosis cases P=0.01 (Fisher’s exact test) DVS: deep venous systems; SVS: superficial venous system

Venous system	Re-exploration (n=4)	No re-exploration (n=65)	Total
DVS	1	57	58
SVS	3	8	11

Single venous anastomosis was not associated in the current study with increased risk of re-exploration compared with multiple venous anastomosis (odds ratio: 0.93, 95% CI: 0.22 to 3.9, p=1.00 by Fisher’s exact test).

## Discussion

The recipient vein system

The choice of recipient vein is an important factor in the outcome of head and neck free tissue reconstruction. Approximately half of all cases of thrombosis in the literature were attributed to the venous system [3,17]. Recipient veins with a lower risk of venous thrombosis should therefore be selected. Thrombosis in blood vessels might be induced by kinking or compression of blood vessels. The SVS is located more superficially than the DVS, and hence it is thought to be comparatively more susceptible to cervical torsion and compression. Twisting, kinking, stretching, or compression of the vein have been reported as a cause of venous thrombosis, indicating that it has a greater effect during intraoperative closure than postoperative neck movement [18]. These factors can be controlled to some extent by intraoperative fixation of the vascular arrangement with fibrin glue and postoperative neck rest. In this study, the increased risk of reoperation for the SVS system possibly resulted from the algorithm used at our institution for the selection of recipient veins. The IJVS is our first choice of recipient vein due to the advantages of recipient vein preparation. The SVS is selected when internal jugular vein thrombosis is expected or when the internal jugular vein cannot be utilized due to radical neck dissection. In other words, when the SVS is utilized as the single recipient vein at our institution, it is likely that highly invasive manipulation of the cervical vessels is performed. Contralateral venous anastomosis with vein grafting could be considered in these cases.

Single vs. multiple anastomosis

In the current study, double anastomosis did not decrease the risk of re-exploration. However, considering the low rate of complications, it is premature to argue that double anastomosis is unnecessary based on these results alone. Meta-analyses and multicenter studies are required due to the limited amount of data available from a single institution. Depending on the condition of the recipient veins and the vasculature of the individual flap, a second anastomosis is performed whenever possible at our institution.

Flap types

As shown in Table 2, the re-exploration rate for the SCIP flap was higher than for other flaps, except for the scapular flap, of which there were very few cases. This outcome may be attributed to the characteristics of the SCIP flap, such as its small vessel caliber and technically demanding microvascular anastomosis, and its susceptibility to twisting, kinking, stretching, or compression due to its low perfusion volume. The use of the superficial venous system may have compounded these issues. A dedicated analysis of the SCIP flap with a larger case series is required to clarify these findings.

Patient-related factors

Factors involved in thrombosis after free flap reconstruction have been examined in several studies. In a study focusing on hemoglobin and hematocrit, there was an increased risk of total flap necrosis with preoperative hemoglobin levels <10 g/dL and hematocrit <30% [19]. The study included cases of multiregional reconstruction, such as limb trauma, and the hematological status increased the risk in the subset analysis focused on trauma cases. In the current study, we focused on head and neck reconstruction, and hemoglobin and hematocrit levels were not significantly different between those with and those without re-exploration. This could be because head and neck reconstruction is a planned surgery, and cases with extremely low hemoglobin and hematocrit are excluded. We found no significant differences in patient factors between cases with and without re-exploration. These factors may therefore influence the general postoperative condition, but not re-exploration.

Limitations of the study

This study has several limitations. Firstly, the low overall re-exploration rate resulted in insufficient statistical power, particularly for the subgroup analyses. Second, the retrospective design is inherently susceptible to selection bias and challenges posed by missing data, which can limit the scope of analysis. Multicenter prospective studies are therefore required to gain deeper insights into the topic.

## Conclusions

Our findings suggest that the use of SVS as a single recipient vein increases the risk of re-exploration following head and neck free flap reconstruction. This increased risk is likely attributable to the vein's vulnerability to intraoperative and postoperative compression. However, since this was a single-institution-based study with a low complication rate, the generalizability of these findings is limited.
